# Anti‐LGI1, anti‐GABABR, and Anti‐CASPR2 encephalitides in Asia: A systematic review

**DOI:** 10.1002/brb3.1793

**Published:** 2020-08-12

**Authors:** Prinska Ghimire, Ujjwal Prakash Khanal, Bikram Prasad Gajurel, Ragesh Karn, Reema Rajbhandari, Sunanda Paudel, Niraj Gautam, Rajeev Ojha

**Affiliations:** ^1^ Maharajgunj Medical Campus Institute of Medicine Kathmandu Nepal; ^2^ Department of Neurology Tribhuvan University Teaching Hospital Kathmandu Nepal

**Keywords:** anti‐CASPR2 encephalitis, anti‐GABABR encephalitis, anti‐LGI1 encephalitis, autoimmune encephalitis

## Abstract

**Aim:**

We aim to review the literature to collate and describe features of encephalitides arising from autoantibodies against leucine‐rich glioma‐inactivated 1 (LGI1), gamma aminobutyric acid receptor (GABABR), and contactin‐associated protein‐like 2 (CASPR2) in Asian populations and compare them with findings of Western studies.

**Methods:**

Peer‐reviewed articles published till 24 May 2020 were searched, and original, full‐text studies from Asia with serum/CSF antibody‐based diagnosis and at least 2 patients were selected. Twenty‐four studies with 263 patients (139 anti‐LGI1, 114 anti‐GAGABR, and 10 anti‐CASPR2) were included. Data were pooled to produce descriptive information on demographics, clinical characteristics, diagnostics, treatments, and outcome.

**Results:**

The mean age was 54.2 (anti‐LGI1), 55.2 (anti‐GABABR), and 47.7 years (anti‐CASPR2), with an overall male predominance of 62.0%. Commonest clinical features across all types were seizures (87.5%), memory deficits (80.7%), psychiatric disturbances (75.9%), and altered consciousness (52.9%). Four anti‐LGI1, 40 anti‐GABABR, and 1 anti‐CASPR2 patients had tumors. CSF, MRI, and EEG were abnormal in 33.3%, 54.1%, and 75% patients in anti‐LGI1; 60.0%, 49.6%, and 85.7% in anti‐GABABR; and 50%, 44.4%, and 100% in anti‐CASPR2 patients, respectively. 95.6% patients received first‐line therapy alone (steroids/IVIG/Plasma therapy), and 4.4% received second‐line therapy (rituximab/cyclophosphamide). 91.7%, 63.6%, and 70% of patients had favorable outcomes (modified Rankin Score 0–2) with mortality rates at 2.5%, 23.2%, and 0% in the three types, respectively.

**Conclusion:**

Our findings suggest that these disorders present in Asian patients at a relatively young age often with features of seizures, memory deficits, and psychiatric disturbances and usually demonstrate a favorable clinical outcome.

## Graphical abstract

In this article, we aim to review the literature to collate and describe features of encephalitides arising from autoantibodies against leucine‐rich glioma‐inactivated 1 (LGI1), gamma aminobutyric acid receptor (GABABR), and contactin‐associated protein‐like 2 (CASPR2) in Asian populations and compare them with findings of Western studies.

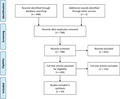

## 1 INTRODUCTION

The term “autoimmune encephalitis” encompasses a wide spectrum of disorders characterized by a variety of limbic and extra‐limbic manifestations resulting from immune mediated attack against intracellular and neuronal cell surface antigens (Graus, Saiz, & Dalmau, 2010; Lancaster, Martinez‐Hernandez, & Dalmau, 2011). Disorders targeting intracellular onconeuronal antigens like anti‐Hu encephalitis were initially discovered in the 80s. These mostly present as paraneoplastic disorders and have a T cell‐mediated pathogenesis (Dalmau & Rosenfeld, 2008; Graus et al., 2001). Owing to their paraneoplastic association and suboptimal treatment response, the overall interest shown toward these disorders was limited. Since the early 2000s, however, there has been an increasing recognition of antibodies directed against synaptic and cell surface antigens that are responsive to immunotherapy (Lai et al., 2010; Lancaster et al., 2010; Vincent et al., 2004). These were initially believed to be a rare entity, but the prevalence of these disorders is now being understood to approximate that of infectious encephalitis (Dubey et al., 2018). With increasing recognition of newer antigens and better diagnostic testing, these numbers are expected to be rising globally.

The most commonly described variant is the anti‐N‐Methyl D‐Aspartate Receptor (NMDAR) encephalitis, which accounts for 50% of autoimmune encephalitis cases (Lancaster et al., 2011). Much less is known about other variants, namely the anti‐leucine‐rich glioma‐inactivated 1 (LGI1), anti‐gamma aminobutyric acid‐B receptor (GABABR), and anti‐contactin‐associated protein‐like 2 (CASPR2) encephalitis, whose prevalence has been shown to be significant at 30%, 5%, and 3%, respectively (Lancaster et al., 2011). Further, only a small proportion of studies on them have been conducted in Asia, often with inconsistent findings. In this review, we compile and describe the clinical features, diagnostic findings, treatments, and outcomes of these disorders in Asian population and compare them with the findings of Western studies.

## 2 METHODS

### 2.1 Data collection

Databases such as MEDLINE, EMBASE, and Cochrane were searched for all peer‐reviewed articles published until 24 May 2020 using the keywords “Autoimmune Encephalitis,” “Anti‐LGI1 encephalitis,” “Anti‐CASPR2 encephalitis,” “Anti‐AMPAR encephalitis,” “Anti‐GABABR encephalitis,” and their variants, along with names of all Asian countries and capitals connected with "OR" and "AND" Boolean operators. In addition, names of the most populous cities in respective countries were also used for the search (details in Appendix S1). Further, references of collected articles were consulted for finding studies not identified in the original search. In case of partial availability of information, authors of concerned articles were contacted via email.

### 2.2 Eligibility criteria

Original research articles including information on clinical features, treatment, and/or its response for at least one of the four common variants of autoimmune encephalitides after anti‐NMDAR encephalitis, that is, anti‐LGI1, anti‐CASPR2, anti‐GABABR, and anti‐α‐amino‐3‐hydroxy‐5‐methyl‐4‐isoxazolepropionic acid receptor (anti‐AMPAR) encephalitis were considered eligible for inclusion. Only studies conducted in Asia having definite diagnosis with laboratory confirmation (CSF/serum antibody detection) were included.

The following exclusion criteria were applied:

1. Single case reports,
2. Inadequate or unclear descriptions,
3. Cases described under a wider set of diseases where necessary information could not be isolated,
4. Full text unavailable,
5. Not in English.

**FIGURE 1:**
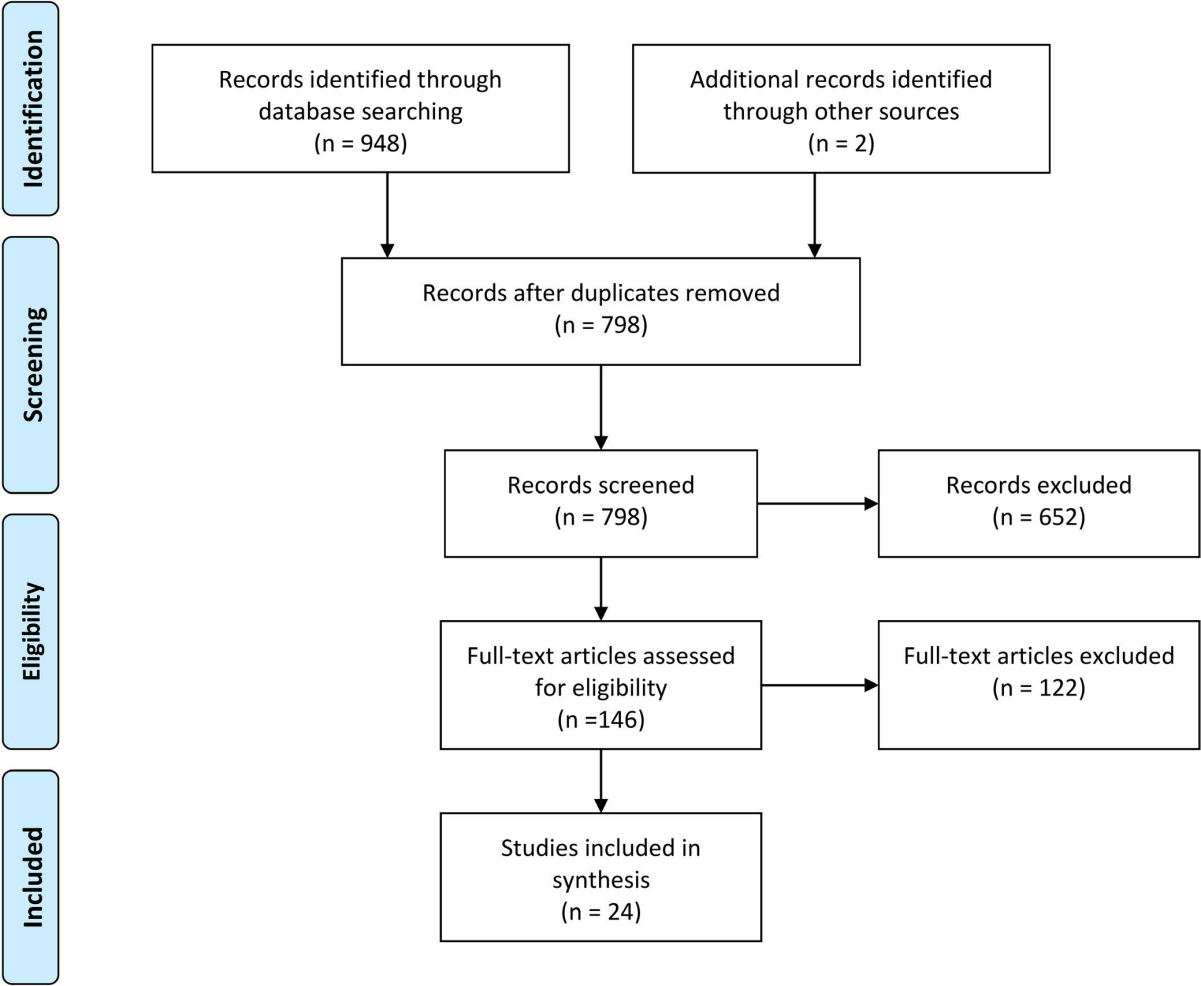
PRISMA flowchart showing the selection process for included studies

For multiple studies including a common set of patients, the study with the greater number of patients in total was selected. Any difference of opinion regarding eligibility was resolved using a consensus between the authors. The PRISMA diagram detailing the selection process is shown in Figure 1.

### 2.3 Data extraction and management

Authors (UPK and PG) independently performed a detailed review of selected studies and extracted the following information: name of the first author, year of publication, country of study, study design, number of patients, age, sex, type of encephalitis, tumor associations, prodromal symptoms, clinical manifestations, CSF studies, EEG/MRI/brain biopsy findings, additional diagnostic tools used (if any), treatment methods, and outcome measurement. Commonly reported parameters indicating abnormalities seen in encephalitides were recorded for CSF, MRI, EEG, and brain biopsy, or any abnormality if the details were not specified. In recording clinical manifestations, faciobrachial dystonic seizures (FBDS) were recorded under “seizures” category, along with other types of seizures, although a separate record was kept for FBDS as well. Features described as “confusion,” “disorientation,” “altered consciousness,” or “decreased consciousness” were all listed under “altered consciousness.” In treatment, corticosteroids (intravenous, oral), intravenous immunoglobulin (IVIG), and plasma therapy were included in first‐line immunotherapy. Plasmapheresis, plasma exchange, and immunoadsorption were all recorded under “Plasma Therapy” owing to the similarities in their principles. Second‐line therapy included rituximab and cyclophosphamide along with steroid‐sparing agents like azathioprine and mycophenolate mofetil. Outcomes were recorded in terms of modified Rankin Scale (mRS) scores whenever applicable, and any other scales used were additionally recorded. Discrepancies were resolved by consulting the third expert reviewer (RO). The information was entered using a piloted form and subsequently recorded in Microsoft Excel 2013 (Microsoft Corp, Redmond, USA).

### 2.4 Analysis

The recorded data were pooled to produce information on the demographics, clinical characteristics, diagnostics, and the treatment modalities in descriptive terms of means, frequencies and proportions. Both qualitative and quantitative assessments were made whenever suitable. For quantitative synthesis, each data point of interest was counted in the numerator if it was mentioned to be present in the paper, and values were only added to the denominator if the study explicitly implied that an attempt/investigation had been made to explore the data point in question. Studies using any additional feature as an inclusion criterion were excluded from quantitative analysis regarding the feature in question and its associated variables.

## 3 RESULTS

### 3.1 Search results

Using the search parameters, 948 records were identified and two were added after going through the references of selected papers. After removing the duplicates, 798 records were evaluated based on their titles and abstracts using the eligibility criteria, which led to exclusion of a further 652 papers. 146 papers were read fully. Finally, 24 papers that fulfilled our inclusion criteria were included in the study (Bing‐Lei et al., 2019; Chen et al., 2017; Cui et al., 2018; Gao et al., 2016; Guan et al., 2015; Kannoth et al., 2018; Kim et al., 2014; Li, Ma, Zhang, & Lian, 2018; Li, Wu, et al., 2018; Li, Song, Liu, & Wang, 2020; Li, Cui, Shi, & Wang, 2016; Lin et al., 2019; Qiao et al., 2017; Shin et al., 2013; Si, Wang, Liu, Zhang, & Hu, 2019; Sunwoo et al., 2015; Wang, Hao, He, He, & Wang, 2018; Yang, Li, Zhao, Liu, & Wang, 2019; Yeo et al., 2018; Yu, Yu, Fang, Zhang, & Lin, 2016; Zeng, Cao, Zheng, & Yu, 2020; Zhang et al., 2020; Zhao, Zhang, Gao, & Sun, 2016; Zhu et al., 2020) (Figure 1).

### 3.2 Study characteristics

Of the 24 studies included, 8 were cohort studies, the rest being case series. The total number of patients included was 263. The median number of patients per study was 10, ranging from 3 to 28. Most studies were conducted in China (*n* = 19), followed by South Korea (*n* = 3), India (*n* = 1), and Singapore (*n* = 1). The most commonly described variant was anti‐LGI1 encephalitis (13 studies, 139 patients), followed by anti‐GABABR (9 studies, 114 patients), and anti‐CASPR2 encephalitis (3 studies, 10 patients). One paper discussed more than two variants (Yeo et al., 2018). No study examining anti‐AMPAR encephalitis was eligible for the review. Details about selected studies can be found in Tables 1, 2, 3.

**TABLE 1:** Summary of Findings of studies included for anti‐LGI1 encephalitis

Author/Year	No. of patients	Mean/median age	Sex ratio (% male)	Clinical presentation	CSF Abnormality	MRI (abnormality, commonest sites)	EEG abnormality	Outcome (mRs 0–2)	Relapse
Li et al., 2020	9	52	4/5 (44)	Memory dysfunction 100%, FBDS 44%, Psychiatric symptoms 100%, Sleep disorders 66%, Hyponatremia 78%	22%	66%, Hippocampus, BG	9/9 (100)	9/9 (100)	N/A
Yang et al., 2019	24	56.9	20/4 (83)	Memory dysfunction 75%, FBDS 38%, Psychiatric symptoms 58%, Hyponatremia 58.3%	29%	29%, MTL	22/24 (91)	24/24 (100)	3/24 (13)
Li, Wu, et al., 2018	19	58	15/4 (79)	Memory dysfunction 69%, FBDS 69%, Psychiatric symptoms 74%, Hyponatremia 26.3%	74%	26%, BG, Hippocampus	11/17 (65)	18/19 (95)	6/19 (32)
Li, Wu, et al., 2018	8	63.4	5/3 (62.5)	Memory dysfunction 100%, FBDS 100%, Psychiatric symptoms 88%, Sleep disorders 50%, Hyponatremia 75%	12%	56%, Hippocampus, BG	2/8 (25)	8/8 (100)	2/8 (25)
Yeo et al., 2018	4	74	3/1 (75)	Memory dysfunction 75%, FBDS 50%, Psychiatric symptoms 100%, Sleep disorders 50%, Hyponatremia 25%	N/A	100%, MTL, Hippocampus	3/4 (75)	4/4 (100)	2/4 (50)
Bing‐Lei et al., 2019	3	53	1/2 (33)	Memory dysfunction 100%, FBDS 33%, Psychiatric symptoms 100%, Hyponatremia 100%	0%	100%, Hippocampus, MTL	3/3 (100)	2/3 (67)	N/A
Chen et al., 2017, ^a^	18	47	8/10 (44)	Memory dysfunction 100%, FBDS 66.7%, Disorientation/confusion 94%, Sleep disorders 33%, Hyponatremia 44%	N/A	67%. MTL, BG	18/18 (100)	N/A	N/A
Wang et al., 2018	13	40.5	5/8 (39)	Memory dysfunction 100%, FBDS 100%, Psychiatric symptoms 100%	38%	31%, MTL, Hippocampus	8/13 (62)	10/13 (77)	N/A
Gao et al., 2016	10	51.5	7/3 (70)	Memory dysfunction 90%, FBDS 100%, Psychiatric symptoms 60%, Sleep disorders 20%, Hyponatremia 80%	30%	90%, Hippocampus, BG, MTL	10/10 (100)	9/10 (90)	3/10 (30)
Li et al., 2016, ^a^	10	58.2	6/4 (60)	Memory dysfunction 40%, FBDS 70%, Hyponatremia 50%	0%	60%, MTL	N/A	10/10 (100)	N/A
Zhao et al., 2016	3	54	1/2 (33)	Memory dysfunction 100%, FBDS 66.7%, Psychiatric symptoms 33%, Hyponatremia 100%	33%	100%, BG, MTL	1/3 (33)	2/3 (67)	N/A
Yu et al., 2016	4	54	2/2 (50)	Memory dysfunction 100%, FBDS 50%, Psychiatric symptoms 50%, Hyponatremia 100%	50%	100%, Hippocampus, BG, MTL	0/4 (0)	4/4 (100)	N/A
Shin et al., 2013	14	58.8	8/6 (57)	Cognitive dysfunction 86%, FBDS 71%, Sleep disorders 7%, Hyponatremia 43%	52%	71%, MTL	10/13 (77)	11/14 (79)	2/14 (14)

Note

Data are expressed as proportion (%) unless otherwise specified. Age is expressed in years.

Abbreviations: BG, Basal Ganglia; CSF, Cerebrospinal fluid; EEG, Electroencephalogram; FBDS, Faciobrachial Dystonic Seizures; MRI, Magnetic Resonance Imaging; MTL, Medial Temporal Lobe; N/A, Not available.

^a^ Seizures were additional inclusion criteria in these papers.

**TABLE 2:** Summary of Findings of studies included for anti‐GABABR encephalitis

Author/Year	No. of patients	Mean/Median age	Sex ratio/ (%males)	Clinical presentation	CSF Abnormality	MRI (abnormality, commonest areas)	EEG abnormality	Outcome (mRS 0–2)	Relapse
Zhang et al., 2020	19	58.3	10/9 (53)	Memory dysfunction 95%, Psychiatric symptoms 89%, Seizures 90%	NA	72%, Hippocampus, MTL	17/19 (89)	11/19 (58)	3/19 (16)
Zhu et al., 2020	14	52	9/5 (64)	Memory dysfunction 79%, Psychiatric symptoms 64%, Seizures 100%	NA	57%, Hippocampus, temporal lobe	10/14 (71)	N/A	N/A
Lin et al., 2019	28	53	17/11 (61)	Memory dysfunction 93%, Psychiatric symptoms 86%, Seizures 96%	NA	25%, Hippocampus, MTL	18/24 (75)	16/28 (57)	6/28 (21)
Zeng et al., 2020	7	44.7	4/3 (57)	Memory dysfunction 86%, Psychiatric symptoms 71%, Seizures 100%	29%	72%, Hippocampus, MTL	6/6 (100)	4/7 (57)	N/A
Si et al., 2019	5	41.2	3/2 (60)	Confusion and disorientation 60%, Psychiatric symptoms 100%, Seizures 80%	0%	60%, Hippocampus, MTL	5/5 (100)	5/5 (100)	N/A
Cui et al., 2018	11	63	7/4 (64)	Memory dysfunction 100%, Psychiatric symptoms 46%, Seizures 91%	73%	36%, Hippocampus, MTL	10/11 (91)	8/11 (73)	N/A
Guan et al., 2015	18	56.4	13/5 (77)	Memory dysfunction 67%, Psychiatric symptoms 61%, Seizures 94%	NA	56%, MTL	12/14 (86)	11/18 (61)	N/A
Qiao et al., 2017	7	51.2	6/1 (86)	Memory dysfunction 100%, Psychiatric symptoms 100%, Seizures 100%	100%	57%, MTL, Hippocampus	7/7 (100)	3/7 (43)	N/A
Kim et al., 2014	5	63.6	2/3 (67)	confusion and disorientation 100%, Psychiatric symptoms 100%, Seizures 60%	80%	40%, MTL, rest non‐specific	5/5 (100)	5/5 (100)	N/A

Note

Data are expressed as proportion (%) unless otherwise specified. Age is expressed in years.

Abbreviations: CSF, Cerebrospinal fluid; EEG, Electroencephalogram; MRI, Magnetic Resonance Imaging; MTL, Medial Temporal Lobe; N/A, Not available.

**TABLE 3:** Summary of findings of studies included for anti‐CASPR2 encephalitis

Author/Year	No. of patients	Mean/Median age	Sex ratio/% male	Clinical presentation	CSF Abnormality	MRI (abnormality, commonest areas)	EEG abnormality	Outcome (mRs 0–2)	Relapse
Yeo et al., 2018	2	63	2/0 (100)	Limbic encephalitis 2/2	None	2/2 (100), B/l MTL hyperintensity (FLAIR)	2/2 (100), Bitemporal Sharp waves	2/2 (100)	2/2 (100)
Kannoth et al., 2018	3	54	2/1 (33)	Seizures, pain, tremor, disorientation	2/2 (100), increased protein	½ (50), small vessel ischemic changes	2/2 (100), mild electric dysfunction	1/3 (33.33)	1/3 (33.3)
Sunwoo et al., 2015	5	37.8	3/2 (60)	Seizures 80%, Insomnia 40%, Neuropathy and pain 40%	2/4 (50), 2 pleocytosis, 1 increased protein	2/5 (40), meningeal enhancement, MTL	5/5 (100), slowing, epileptiform discharges	4/5 (80.0)	N/A

Note

Data are expressed as proportion (%), unless otherwise specified. Age is expressed in years.

Abbreviations: B/l, Bilateral; CSF, Cerebrospinal fluid; EEG, Electroencephalogram; MRI, Magnetic Resonance Imaging; MTL, Medial Temporal Lobe; N/A, Not available.

### 3.3 Demographic and clinical characteristics

The age of the patients ranged from 8 to 85 years, the mean age being 54.4 years. The mean age for the anti‐LGI1 group was 54.17 (range 18–85 years), for anti‐GABABR was 55.2 years (range 18–76 years), and studies with anti‐CASPR2 had the lowest mean age, that is, 47.7 years (range 8–72 years). 62.0% of patients were males (anti‐LGI1 61.2%, anti‐GABABR 62.3%, anti‐CASPR2 70%).

Information regarding tumor association was provided in 23 studies (*n* = 245). Four anti‐LGI1 patients had tumors: 1 patient had lung cancer, 1 colon adenocarcinoma, 1 renal cell carcinoma, and 1 rectal adenocarcinoma. Of the 40 anti‐GABABR patients with tumors, there were 36 cases of lung cancers, 1 thymoma, 1 mediastinal teratoma, 1 pancreatic carcinoma, and 1 cervical carcinoma. One anti‐CASPR2 patient had a pituitary microadenoma. Interestingly, tumors in some patients were detected after they were discharged from the hospital in some anti‐GABABR encephalitis cases (Kim et al., 2014).

The most commonly seen clinical presentation was seizure, followed by memory dysfunction, and psychiatric/behavioral symptoms. Altered consciousness, dysautonomia, speech disorders, and central hypoventilation were also reported in numerous patients, the details of which can be found in Table 4. In the anti‐LGI1 group, memory dysfunction and seizures were the most common symptoms, followed by psychiatric disturbance, altered consciousness, sleep disorders, autonomic dysfunction, and speech disorders. FBDS was a feature reported only in this variant, seen in 66.7% of patients. Generalized tonic clonic seizures (GTCS) were most commonly seen in anti‐LGI1 patients (30/63) after FBDS, along with complex partial seizures now called focal impaired awareness seizures (6/20), simple partial seizures now called focal aware seizures (4/18), status epilepticus (6/43), and mesial temporal lobe seizures (MTLS) (6/9) now also a part of focal impaired awareness seizures. 8/24 cases were reported as new onset refractory seizures. Hyponatremia was present in 56.3% patients.

**TABLE 4:** Demographic and clinical characteristics

	Total	Anti‐LGI1	Anti‐GABABR	Anti‐CASPR2
Number of patients	263	139	114	10
Mean age in years	54.4 ( *n* = 201)	54.17 (*n* = 105)	55.2 (*n* = 86)	47.7 (*n* = 10)
Sex (m/f, %m)	163/100, 62.0	85/54, 61.2	71/43, 62.3	7/3, 70.0
Tumors				
Information available for	245	121	114	10
Total number (% total)	45 (18.4)	4 (3.3)	40 (36.0)	1 (10.0)
Lung Cancers	37	1	36	0
Thymoma	1	0	1	0
Adenocarcinoma	2	2	0	0
Others	5	1	3	1
Clinical features				
Psychiatric and behavioral	180/237 (75.9)	90/115 (78.3)	88/114 (77.2)	2/8(25)
Seizures	204/233 (87.5)	92/111 (82.8)	106/114 (93)	6/8 (75)
Motor Dysfunction	17/122 (13.9)	NA	15/114 (13.2)	2/8 (25)
Memory/Cognitive Dysfunction	209/259 (80.7)	117/139 (84.2)	92/112 (82.1)	0/8(0.0)
Speech disorders	13/261 (5.0)	4/139 (2.9)	7/114 (6.1)	1/8(12.5)
Altered consciousness	108/204 (52.9)	53/96 (55.2)	51/100 (51)	4/8 (50)
Autonomic dysfunction	20/261 (7.7)	10/139 (7.2)	8/114 (7.0)	2/8 (25)
Central hypoventilation	9/261 (3.4)	0(0.0)	9/114 (7.9)	0/8(0.0)
Sleep disorders	35/116 (30.2)	21/63 (33.3)	11/45 (24.4)	3/8 (37.5)
Hyponatremia	74/248 (29.8)	71/126 (56.3)	1/114 (0.9)	2/8 (25)
FBDS	74/233(31.8)	74/111 (66.7)	0 (0.0)	0/8 (0.0)
Prodromal symptoms				
Fever	13	4	8	1
Headache	5	1	4	—
Upper respiratory infections	6	—	6	—
Diarrhea and Vomiting	2	—	2	—
Cold	2	—	2	—
Dizziness	2	1	2	—
CSF antibody positivity	190/212 (89.6)	85/103 (82.5)	103/107 (96.3)	2/2 (100)
Serum antibody positivity	185/199 (93.0)	117/125 (93.6)	64/70 (91.4)	4/4 (100)
CSF Examination				
Abnormality	62/156 (39.7)	39/117 (33.3)	21/35 (60)	2/4 (50)
Pleocytosis	75/239 (39.4)	12/117(10.3)	61/114 (53.5)	2/8 (25)
Increased Protein concentration	64/239 (26.8)	25/117 (21.4)	37/114 (32.5)	2/8 (25)
Oligoclonal bands	15/22 (68.2)	—	15/22 (68.2)	—
EEG findings				
Abnormality	179/222 (80.6)	81/108 (75)	90/105 (85.7)	9/9 (100)
Slowing of activity	105/209 (50.0)	46/95 (48.4)	53/105 (50.5)	6/9 (67.7)
Epileptiform discharges	68/209 (32.5)	25/95 (26.3)	40/105 (38.1)	3/9 (33.3)
MRI findings				
Abnormality	121/233 (51.9)	60/111 (54.1)	56/113 (49.6)	5/9 (44.4)
Medial temporal lobe	69/233 (29.6)	29/111 (26.1)	37/113 (32.7)	3/9 (33.3)
Hippocampus	52/233 (22.3)	23/111 (20.7)	29/113 (25.7)	1/9 (1.1)
Basal Ganglia	12/233 (5.2)	12/111 (10.8)	—	—
Brainstem	3/233 (1.3)	3 (2.2)	—	—
Associated Demyelination	1/233 (0.4)	1 (7.2)	—	—

Note

Data are expressed as proportion (%) unless otherwise stated.

Abbreviations: CSF, Cerebrospinal fluid; EEG, Electroencephalogram; MRI, Magnetic Resonance Imaging.

In anti‐GABABR group, the most frequently reported features were seizures, followed by memory/cognitive dysfunction, psychiatric disturbance, altered consciousness, sleep disorders, autonomic dysfunction, motor disorders, central hypoventilation, and speech disorders. Commonly seen psychiatric alterations were hallucinations, mood disorders, and personality changes. In the anti‐GABABR group, GTCS were the most common seizure type as well (36/48), along with complex partial seizures (now called focal impaired awareness seizures) (9/32), and status epilepticus (22/73). 17/18 were said to have presented with new‐onset refractory seizures. Dysautonomia was usually reported in the form of dysrhythmia, and even diarrhea in one of the patients (Zhang et al., 2020). Movement disorders and cerebellar involvement were reported in five of the studies, manifesting as orofacial dyskinesias, limb involuntary movements, opisthoclonus–myoclonus, and ataxia.

In the 10 anti‐CASPR2 patients, seizures, sleep disorders, motor dysfunction, dysautonomia, and psychiatric disturbances, along with tremors, ataxia, and other cerebellar signs in the form of dysdiadochokinesia were seen. One patient had presented with features of neuromyotonia (Table 4).

Seven studies mentioned the presence of prodromal symptoms. The most commonly reported symptom was fever, followed by headache, upper respiratory tract infections, diarrhea and vomiting, and vertigo.

### 3.4 Diagnostic findings (CSF, EEG, MRI, PET)

#### 3.4.1 Antibody detection

CSF antibodies were positive in 89.6% of patients, whereas 93.0% showed positive titer in the serum. Anti‐LGI1 antibodies were detected in 82.5% in CSF and 93.6% in serum, whereas anti‐GABABR in 96.3% in CSF and 91.4% in serum. Anti‐CASPR2 antibodies were detected using the CSF in 2/2 and serum in 4/4 patients. Only one patient was found to have dual antibody positivity for neuronal cell surface antigens (GABABR and NMDAR). 11/97 anti‐GABAB patients tested positive for classical paraneoplastic antibodies, most of them being anti‐Hu. One anti‐LGI1 patient and seven anti‐GABABR patients had antithyroid antibodies as well.

#### 3.4.2 CSF findings

39.7% patients showed abnormalities in the CSF (33.3% anti‐LGI1, 60% anti‐GABABR, and 50% anti‐CASPR2). CSF pleocytosis and increased protein concentration were the commonest changes (Table 4), including oligoclonal bands in 68.2% of anti‐GABABR patients. Other anomalies reported include alterations in glucose and chloride levels, although they were not consistently mentioned across studies.

#### 3.4.3 EEG findings

EEG was reported to be abnormal in 80.6% of the patients. Findings, however, were reported as non‐specific slowing and epileptiform discharges. 75% of anti‐LGI1 group had EEG abnormalities (focal or diffuse slowing in 48.4% and epileptiform discharges in 26.3%). In contrast, 85.7% of anti‐GABABR encephalitis patients had EEG abnormalities (focal or diffuse slowing in 50. 5% and epileptiform discharges in 38.1%). Other reported anomalies were rather uncommon, with the majority being fast waves and sharp waves.

#### 3.4.4 MRI findings

51.9% had abnormal MRI results (54.1% anti‐LGI1, 49.6% anti‐GABABR, and 55.6% anti‐CASPR2). In the anti‐LGI1 group, the commonest sites of lesion (seen on T2/FLAIR) were the medial temporal lobe (MTL) and basal ganglia. In anti‐GABABR encephalitis, lesions were most commonly reported in the MTL and in the hippocampus. However, frontal, temporal, and parietal lobe involvement was also reported in certain studies for both of these conditions (Li, Wu, et al., 2018; Lin et al., 2019; Zeng et al., 2020). Among anti‐CASPR2 patients, three had MTL lesions, one showed meningeal enhancement, and the anomaly was not described in the other.

#### 3.4.5 PET findings

18F‐Fluorodeoxyglucose positron emission tomography (FDG‐PET) was described as a diagnostic tool in 10 of the studies for tumor detection and localization of brain lesions. In anti‐LGI1 encephalitis, the most commonly recorded findings were hypermetabolism in the basal ganglia, amygdala, and cortex. A total of 90% abnormality rate was reported in one of the studies against a 71% abnormality rate in MRI, while another showed a rate of 60% against a 26% MRI abnormality detection (Li, Wu, et al., 2018; Shin et al., 2013). MTL and diffuse cortical hypermetabolism were the most commonly noted lesions in anti‐GABABR patients. Kim T reported a higher sensitivity of FDG‐PET to MRI (3/5 versus. 2/5) in anti‐GABABR encephalitis (Kim et al., 2014). Interestingly, PET scan reports were available for three patients with anti‐CASPR2 encephalitis, all of whom showed normal findings.

Brain biopsy was not used as a tool for diagnosis in any of the patients included.

#### 3.4.6 Treatment methods and outcomes

First line of therapy alone was used in 95.6% of the patients (Table 5). Corticosteroid alone was used in 35.6% (40.74% anti‐LGI1, 28.3% anti‐GABABR, and 44.44% anti‐CASPR2). 14.4% received IVIG alone (5.19% anti‐LGI1 and 27.36% anti‐GABABR), and 45.2% received a combination of corticosteroids and IVIG (48.89% anti‐LGI1, 42.45% anti‐GABABR, and 22.22% anti‐CASPR2). Only one LGI patient received plasma therapy combined with corticosteroids and IVIG (Shin et al., 2013). Second‐line therapy was used with the first line in 3.2% (2.96% anti‐LGI1, 1.9% anti‐GABABR, and 22.22% anti‐CASPR2). Rituximab was used in three anti‐LGI1 patients, whereas cyclophosphamide in one anti‐LGI1, two anti‐GABABR, and two anti‐CASPR2 patients. Azathioprine was used in two anti‐LGI1 patients with first‐/second‐line treatment, whereas mycophenolate mofetil was used in one anti‐CASPR2 patient. Two anti‐LGI1, six anti‐GABABR, and one anti‐CASPR2 patients did not receive any immunotherapy.

**TABLE 5:** Treatment and outcome

	Total	Anti‐LGI1	Anti‐GABABR	Anti‐CASPR2
First‐line treatment	239/250 (95.6)	129/135 (95.6)	104/106 (98.1)	6/9 (66.7)
CS alone	89 (35.6)	55 (40.7)	30 (28.3)	4 (44.4)
IVIG alone	36 (14.4)	7 (5.2)	29 (27.4)	—
CS + IVIG	113 (45.2)	66 (48.9)	45 (42.5)	2 (22.2)
Plasma alone	0	—	—	—
Plasma therapy + CS/IVIG	1 (0.4)	1 (0.7)	—	—
Second‐line treatment				
Used alone	0	—	—	—
With 1st line	8 (3.2)	4 (3.0)	2 (1.9)	2 (22.2)
Rituximab + 1st line	3 (1.2)	3 (2.2)	—	—
Cyclophosphamide + 1st line	5 (2)	1 (0.7)	2 (1.9)	2(22.2)
Azathioprine + 1st/2nd line	2 (0.8)	2 (1.48)	—	—
Mycophenolate mofetil + 1st/2nd line	1 (0.4)	—	—	1 (11.11)
Anti‐epileptics	132/142 (93.0)	81/90 (90.0)	49/49 (100)	2/3 (66.7)
Timing of outcome assessment (months)	12.5 (*n* = 173)	7.9	14.1	9.4
Mean duration of hospitalization (in days)	27.61	27.86	27	—
Relapse				
Yes	30/112 (26.8)	18/79 (22.8)	9/47 (19.2)	3/5 (60.0)
No	82/112 (73.2)	61/79 (77.2)	38/47 (80.9)	2/5 (40.0)
Outcome (in median mRS)				
CS alone	1 (*n* = 34)	1 (*n* = 13)	1 (*n* = 17)	1.5 (*n* = 4)
IVIG alone	2 (*n* = 18)	1.5 (*n* = 4)	1 (*n* = 14)	—
CS + IVIG	1 (*n* = 43)	0 (*n* = 17)	1.5 (*n* = 24)	0.5 (*n* = 2)
1st + 2nd line	2 (*n* = 3)	2 (*n* = 3)	—	—
Azathioprine/Mycophenolate mofetil + 1st/2nd line	1 (*n* = 3)	3 (*n* = 2)	—	1 (*n* = 1)
Overall outcome in mRS				
Information available for	230	121	99	10
0–2	181 (78.7)	111 (91.7)	63 (63.6)	7 (70.0)
3–4	14 (6.9)	4 (3.3)	9 (9.1)	1 (10.0)
5	5 (2.2)	1 (0.8)	4 (4.0)	—
6	26 (11.3)	3 (2.5)	23 (23.2)	—
N/A	4 (1.7)	2 (1.7)	—	2 (20.0)
Total Mortality	26/230 (11.3)	3/121 (2.5)	23/99 (23.2)	0/10 (0)
Tumor progression	13 (50)	—	13 (56.5)	—
Pulmonary infection (e.g. Pneumonia)	4 (15.4)	1 (33.3)	3 (13.0)	—
Respiratory failure	4 (15.4)	—	4 (17.4)	—
Status epilepticus	3 (11.5)	1 (33.3)	2 (8.7)	—
Septic shock	1 (3.9)	—	1 (4.4)	—
Not mentioned	1 (3.9)	1 (33.3)	—	—

Note

Data are expressed as proportion (%) or *n* (%) unless otherwise stated.

Abbreviations: CS, Corticosteroids; IVIG, Intravenous Immunoglobulin; mRs, Modified Rankin Score.

Most common symptomatic treatment was the administration of anti‐epileptics, followed by antipsychotics. Anti‐epileptics were given to 93% of the patients. Most papers did not mention the use of maintenance therapy. Only 33 out of 37 patients (23/25 anti‐LGI1, 7/7 anti‐GABABR, and 3/5 anti‐CASPR2) received maintenance therapy either in the form of steroids or steroid‐sparing agents or both, steroids being preferred the most. The mean duration of hospitalization was 27.6 days. Out of data available for 46 anti‐GABABR patients, seven were admitted to the Intensive Care Unit (ICU). The commonest complications reported during hospital admission were pneumonia, respiratory failure, urinary tract infection (UTI), and deep vein thrombosis (DVT).

Among the 36 anti‐GABABR patients with lung cancer, nine received radiation therapy with chemotherapy, four received only chemotherapy, two received surgery with chemotherapy, four refused all tumor treatment methods, and treatment was not mentioned for the remaining 17 patients. One anti‐GABABR patient with thymoma underwent surgery. Tumor management was not mentioned in the remaining nine patients with other tumors.

Out of 112 patients, 26.8% relapsed (60.0% anti‐CASPR2, 22.8% anti‐LGI1, and 19.2% anti‐GABABR). Among 26 patients treated with corticosteroids alone, six patients relapsed (2/17 anti‐LGI1, 1/4 anti‐GABABR, and 2/2 anti‐CASPR2), whereas seven patients treated with combined corticosteroids and IVIG relapsed (*n* = 31, 5/24 LGI1, and 2/7 GABA). There was no relapse in four anti‐GABABR patients treated with IVIG alone. Relapse was reported in one patient treated with rituximab along with corticosteroids, IVIG, and plasmapheresis, whereas one patient treated with rituximab, corticosteroids, and IVIG did not relapse (Shin et al., 2013). A patient treated with azathioprine and corticosteroid relapsed, whereas a patient treated with azathioprine combined with both the first and second lines did not relapse (Shin et al., 2013). Among the two patients given corticosteroids combined with cyclophosphamide, one patient relapsed (Kannoth et al., 2018).

The mean timing of outcome assessment was 12.51 months (Table 5). Though the majority measured outcomes in terms of mRS, few studies simply defined it as “improved/recovered” or not, discarding any objective assessment. The median mRS was 1 after treatment with corticosteroids alone, 2 after IVIG alone, 1 after corticosteroids combined with IVIG, and 2 after plasma therapy with corticosteroids/IVIG. The median mRS after treatment with both first‐ and second‐line therapy was 2. The median mRS was 3 for all anti‐LGI1 patients treated with azathioprine combined with the first/second line. Overall, out of 230 patients whose mRS scores were mentioned, 78.7% had mRS 0–2 (91.7% anti‐LGI1, 63.6% anti‐GABABR, and 70% anti‐CASPR2), 6.9% had mRS 3–4 (3.3% anti‐LGI1, 9.1% anti‐GABABR, and 10% anti‐CASPR2), 2.2% had mRS 5 (0.8% anti‐LGI1 and 4.0% anti‐GABABR), and 11.3% were dead, that is, mRS 6 (2.5% anti‐LGI1 and 23.2% anti‐GABABR). One anti‐LGI1 patient declined any immunotherapy, yet had mRS score improved to 1, whereas one anti‐LGI1 patient showed clinical improvement after anti‐epileptics alone (Li et al., 2020; Yeo et al., 2018). Two anti‐LGI1 patients, who were both initially treated with corticosteroids alone, had three relapses. Rituximab and tacrolimus were added that led to cessation of further relapse in one patient (Shin et al., 2013). One anti‐GABABR patient improved even without immunotherapy (Lin et al., 2019). One anti‐CASPR2 patient who refused immunotherapy had no clinical improvement till the latest follow‐up (Sunwoo et al., 2015). Complete seizure control was achieved in 3/4 anti‐CASPR2 patients, with reduction in the remaining one (Sunwoo et al., 2015). Memory impairments, spatial disorientation, apathy, and sleep disorders were the commonly found residual symptoms in anti‐LGI1 patients, whereas memory impairments and seizures were common in anti‐GABABR patients.

The total mortality rate was 11.3% (*n* = 230). Anti‐GABABR had the highest mortality rate of 23.2%, the most common cause being tumor progression (56.5%), followed by pulmonary infections (13.0%), respiratory failure (17.4%), status epilepticus (8.7%), and septic shock (4.4%). Anti‐LGI1 patients had a 2.5% mortality rate, resulting from pulmonary infection (33.3%) and status epilepticus (33.3%). Anti‐CASPR2 patients did not have any associated mortality.

## 4 DISCUSSION

### 4.1 Anti‐LGI1 encephalitis

This disorder was first described in 2001 as anti‐VGKC encephalitis causing limbic symptoms (Buckley et al., 2001). The initial targets for the associated limbic encephalitis were believed to be the Kv1.1 and Kv1.2 subunits of VGKC (Kleopa, Elman, Lang, Vincent, & Scherer, 2006). However, Lai et al. in 2010 showed that the target in fact was the LGI1 (and CASPR2) protein associated with VGKC, not VGKC itself (Lai et al., 2010). LGI1 is a secreted protein ligand expressed primarily in the hippocampus, and its loss has shown to cause hippocampal hyperexcitability leading to fatal epilepsies in mice (Fukata et al., 2006, 2010). In humans, mutations in the LGI1 gene have been reported to be associated with autosomal dominant partial epilepsy with auditory features (Kalachikov et al., 2002). These features are reflected in anti‐LGI1 encephalitis as well.

Multiple Western studies have reported the mean age of patients to be in the range of 60–65 years (Ariño et al., 2016; Celicanin et al., 2017; Finke et al., 2017; Irani et al., 2008; Lai et al., 2010). In contrast, the mean age of patients in our pool was 54.2 years, with some reporting a mean age as low as 40.5 years. Only 2 out of 13 selected studies reported a mean age greater than 60 (Li, Wu, et al., 2018; Yeo et al., 2018). This hints toward a lower age of onset in Asian populations than previously described which could be attributed to possible ethnic/genetic variations or the overall young demographic in these countries. A male predominance was found with 61.2% of all patients being male, which is consistent with previous studies (Ariño et al., 2016; Finke et al., 2017; van Sonderen, Thijs, et al., 2016).

All the patients in the study had presented with limbic encephalitis. Epilepsy and cognitive impairment were the commonest modes of presentation. Seizures were seen in 84.2% patients, in line with Western studies that report their prominence in 75%–100% of patients (Ariño et al., 2016; Celicanin et al., 2017; Irani et al., 2008; Lai et al., 2010). Cognitive impairment was mostly observed as memory deficits in the majority (82%), which accords to that initially reported by Irani et al. (2008), but is lower than many other studies conducted since (Ariño et al., 2016; Finke et al., 2017; van Sonderen, Thijs, et al., 2016). The difference could be attributed to the variation in reporting nomenclature used across studies. Li et al, who reported memory deficits in only 40% of patients, have therefore suggested testing for anti‐LGI1 antibodies even in the absence of this symptom (Li et al., 2016).

FBDS is considered a pathognomonic feature of anti‐LGI1 encephalitis. It is described as brief, very frequent (50 times a day on average) involuntary movements of the arm and ipsilateral face often associated with loss of consciousness, and has also been known to precede cognitive impairment in anti‐LGI1 patients by a considerable duration (median lag of 36 days; Andrade, Tai, Dalmau, & Wennberg, 2011; Irani et al., 2008, 2011, 2013). Its incidence has been reported in around half the patients in previous studies, but was considerably higher at 66.7% in ours, with eight out of 13 studies reporting it in more than 65% of patients (Finke et al., 2017; van Sonderen, Thijs, et al., 2016). While this could have resulted from variations in observation and reporting across studies, the possibility of different disease presentations in different ethnicities cannot be ruled out. Since the presence of FBDS strongly implies the presence of anti‐LGI1 antibodies, this recognition of their higher incidence could potentially lead to earlier diagnosis, treatment and better patient outcomes in Asian populations (Irani et al., 2013).

Hyponatremia, a commonly described symptom resulting from effects of anti‐LGI1 antibodies on hypothalamus and kidneys, was present in 56.3% of our patients, in line with previous studies (Ellison & Berl, 2007). Sleep disorders were seen in 33.3%, also corresponding to previous studies (Ariño et al., 2016; Finke et al., 2017). Features like dysautonomia and speech disorders were less common. Anti‐LGI1 encephalitis has a low tumor concurrence rate of less than ten percent, the majority being thymomas. This is agreed upon by our study, with 3.3% of patients having a tumor, though none being thymomas (Irani et al., 2010).

Anti‐LGI1 antibodies are often found to have a higher titer and detection rate in the serum than in the CSF (Ariño et al., 2016; Vincent et al., 2004). Our study agrees with this, with an 82.5% CSF positivity rate against 93.6% in the serum. We thus suggest serum tests to be considered before repeated lumbar punctures for antibody detection. CSF parameters have been generally described to be in the normal range in the majority of patients (Lai et al., 2010). A third of the patients showed abnormalities in CSF, with 21% having increased protein concentration and 10% with CSF pleocytosis, which is in line with the findings of a recent systematic analysis (Blinder & Lewerenz, 2019). Other reported anomalies in our study included changes in the CSF glucose and chloride levels.

EEG findings were abnormal in 86% in a Danish study, which corresponds with our findings (75%; Celicanin et al., 2017). Focal or diffuse slowing and epileptiform discharges were the commonest anomalies, similar to previous studies (Finke et al., 2017; van Sonderen, Thijs, et al., 2016). However, the findings were non‐specific and not capable of detecting the pathognomonic FBDS. MRI findings have also been reported to be normal in a quarter of patients (van Sonderen, Thijs, et al., 2016). Commonly detected abnormalities are seen in T2/FLAIR weighted images in the MTL and the hypothalamus (Irani et al., 2008; Lai et al., 2010). Basal ganglia lesions in T1 weighted image can be clinically useful in the early FBDS stage (Flanagan et al., 2015). The rate of abnormality was lower in our study (54.1%), which could be because of inconsistent reporting times across studies and lack of adequate follow‐up. Involvement was also seen in the frontal and temporal lobes, suggesting anti‐LGI1 encephalitis attacks multiple areas of the brain beyond the limbic system (Chen et al., 2017). Moribeli et al suggested considering FDG‐PET scans as a potential diagnostic tool that shows basal ganglia hypermetabolism in the early FBDS stages before the MRI can detect it (Morbelli, Djekidel, Hesse, Pagani, & Barthel, 2016). This was supported by our studies, some of which reported a higher sensitivity rate for PET scans compared to MRI (Li, Wu, et al., 2018; Shin et al., 2013).

Studies have demonstrated anti‐LGI1 encephalitis to be fairly responsive to immunotherapy (Irani et al., 2008, 2011; Quek et al., 2012; Vincent et al., 2004). Due to lack of the standard evidence‐based immunotherapy protocols, the absolute order of use of first line and second line and combination of regimens has not been established. In our study, most of the patients were given corticosteroids combined with IVIG (48.89%), which agrees with other Western studies (Ariño et al., 2016; van Sonderen, Thijs, et al., 2016). Overall, steroids were the most commonly preferred immunotherapy (89.6%), corresponding to the most studies (Lai et al., 2010; van Sonderen, Thijs, et al., 2016). Additionally, almost 90% patients received anti‐epileptics in the course of their treatment, reflecting seizure as the commonest presenting manifestation of the disease.

91.74% had a favorable outcome (mRS 0–2), which despite the variabilities in the assessment scale and the duration of follow‐up, fairly corresponds to previous studies (Lai et al., 2010). This significantly favorable outcome could be accredited to the benign natural course of the disease (Szots et al., 2014). The mortality rate in our pool was found to be considerably lower (2.5%) than most studies, possibly attributable to the shorter mean duration of follow‐up (8 months) Further studies with long‐term follow‐up are warranted to explore relatively lower mortality in the Asian population.

Among 95.6% patients treated with the first line, those treated with combined corticosteroid and IVIG and those with corticosteroid alone had better outcomes in terms of median mRS than those treated with IVIG alone. Interestingly, those who were given a combination of first and second line had poorer outcomes and more relapses than those treated with only the first line. This could be attributed to the comparatively lower number of patients receiving combination of first‐ and second‐line therapy (3.0%) and severity bias, alluding to the fact that the second line was added to the first line only in those with severe disease progression. 22.8% of the patients had relapse, which is in accordance to previous work by Quek et al., but in the upper range when compared to other studies (Irani, Gelfand, Bettcher, Singhal, & Geschwind, 2014; Lai et al., 2010; Malter et al., 2014; Quek et al., 2012). This could be linked to the variability of the follow‐up periods. However, the possibility of increased recurrence in the Asian population cannot be discarded. The rate of relapse was similar in those given corticosteroids alone and those given corticosteroids combined with IVIG.

In two patients, who had three relapses and were both initially treated with corticosteroids alone, addition of rituximab and tacrolimus led to cessation of further relapse in one of the two patients (Shin et al., 2013). This further advocates the mitigating role of rituximab in anti‐LGI1 encephalitis, especially in the relapsing cases, both in terms of improving mRS score and maintaining long‐term disease remission (Brown et al., 2014; Irani et al., 2014; Nepal et al., 2020). Additionally, this also suggests that second‐line immunotherapy should be immediately started in those who fail to respond or deteriorate during first‐line immunotherapy, similar to anti‐NMDAR encephalitis (Titulaer et al., 2013).

Mild memory impairments, spatial disorientation, apathy, and sleep disorders are the commonly reported residual symptoms, which has been supported by our study (Malter et al., 2014; van Sonderen, Thijs, et al., 2016). This underlines the importance of early immunotherapy especially during the FBDS stage to delay or even prevent progression to cognitive impairment (Irani et al., 2011; Shin et al., 2013; Thompson et al., 2018). Therefore, early identification and initiation of immunotherapy is warranted to prevent the cognitive deficit and thereby modulate a better long‐term prognosis.

### 4.2 Anti‐GABABR encephalitis

GABA is an important inhibitory neurotransmitter that modulates synaptic excitability and plasticity when it acts via metabotropic GABAB receptor (Benarroch, 2012). GABABR are principally distributed in the cerebral cortex, thalamus, hippocampus, cerebellum, and amygdala (Benarroch, 2012). Studies have shown that mice lacking functional GABABR have spontaneous seizures leading to premature death, behavioral abnormalities, cognitive deficits, and increased locomotor activities (Prosser et al., 2001; Schuler et al., 2001). Similar limbic symptoms were also observed in patients with antibodies directed to GABABR, establishing the pathogenicity (Lancaster et al., 2010).

The average age of onset for anti‐GABABR encephalitis has been reported to be in the range of 60–70 years (Boronat, Sabater, Saiz, Dalmau, & Graus, 2011; Dogan Onugoren et al., 2015; Lancaster et al., 2010; Maureille et al., 2019). The mean age in our pool of patients however was 55.2 years (Kim et al., 2014; Li, Wu, et al., 2018). Only two papers reported a mean age greater than 60 (Cui et al., 2018; Kim et al., 2014). Similar to anti‐LGI1, it is tempting to speculate a lower age of onset of limbic encephalitis in Asian patients. The sex ratio (62.3% males) closely mirrored Western studies (Höftberger et al., 2013; Lancaster et al., 2010).

Anti‐GABABR encephalitis often manifests as limbic encephalitis with early and prominent seizures followed by psychiatric symptoms, disorientation, and memory deficits, which precede a period of recovery (Lancaster et al., 2010; Maureille et al., 2019). Seizures were described as the initial symptom in the majority of patients with 93% experiencing seizures at some point during the disease, followed by cognitive dysfunction and psychiatric manifestations, which agrees with Western papers (Boronat et al., 2011; Dogan Onugoren et al., 2015; Höftberger et al., 2013). Altered consciousness, although reported less than that in aforementioned studies, was one of the commonest manifestations nonetheless. Sleep disorders, central hypoventilation, dysautonomia, and movement disorders were also described, albeit less frequently.

Anti‐GABABR encephalitis is associated with a high tumor concurrence rate of 50%–80% (Boronat et al., 2011; Höftberger et al., 2013; Lancaster et al., 2010). In our study, however, only 36% of the patients were described as having some form of malignancy. This might reflect the relatively younger cohort and/or potential inadequacies in screening and follow‐up time in studied patients. 90% of the tumors were lung cancers, consistent with previous studies (Höftberger et al., 2013; Lancaster et al., 2010). One of the included studies reported tumor detection in some patients after they were discharged, which highlights the need for close follow‐up as suggested by the European Federation of Neurological Societies (EFNS) task force in 2010 (Kim et al., 2014; Titulaer et al., 2013).

Antibodies against GABABR were detected in both CSF (96.3%) and serum (91.4%) in our pool. The rate of CSF abnormality varied widely across selected studies, with a total of 60% patients showing abnormal results, slightly less than that reported in previous works (Dogan Onugoren et al., 2015; Höftberger et al., 2013; Lancaster et al., 2010). A 2019 analysis reported anti‐GABABR encephalitis to have one of the highest rates for pleocytosis, increased protein concentration, and oligoclonal bands out of ten AE variants studied, which was well mirrored in our study (Blinder & Lewerenz, 2019).

Our study showed that MRI, although a useful investigation, is far from reliable as stated in numerous previous reports (Höftberger et al., 2013; Lancaster et al., 2010; Maureille et al., 2019). Almost half the patients had normal MRI; the commonest sites of lesion were the MTL and the hippocampus in T2/FLAIR‐weighted images. Studies have shown MRI findings to vary along the course of the disease, many included studies not mentioning the timings of the investigation could have possibly led to these results (Heine et al., 2015). EEG was more sensitive, detecting abnormalities in 86% patients, which is in accordance with past studies (Lancaster et al., 2010; Maureille et al., 2019). The findings, however, were non‐specific. As such, there has been a growing acceptance of FDG‐PET as a new diagnostic tool for limbic encephalitis (Morbelli et al., 2016). In our study, the commonest PET findings were diffuse cortical and medial temporal lobe hypermetabolism. This was described to be clinically more sensitive than MRI in one of the included studies (Kim et al., 2014). Further research is needed to elucidate its relevance and utility in case of anti‐GABA encephalitis specifically.

Anti‐GABABR encephalitis has shown a far better response when compared to other intracellular antigen antibody‐related limbic encephalitis (Höftberger et al., 2013; Lancaster et al., 2010). Since studies have demonstrated that around 60%–80% of the patients respond well to immunotherapy, it is the most extensively used approach (Höftberger et al., 2013; Jeffery et al., 2013; Lancaster et al., 2010). Anti‐GABABR encephalitis has been reported to cause seizures refractory to anti‐epileptics (Dubey et al., 2018). Congruently, 85.7% patients in our study who were resistant to anti‐epileptics showed plausible responses to immunotherapy. Steroids were administered in 70.8% of the patients and IVIG in 69.8%, corresponding to previous studies (Boronat et al., 2011; Höftberger et al., 2013). However, the optimal treatment regimen has been shown to rely more on the management of the tumor than anything else considering the frequent tumor association (Höftberger et al., 2013; Jeffery et al., 2013; Lancaster et al., 2010). Chemotherapy, which remains the mainstay for the management of small cell lung cancer (SCLC) irrespective of its association with anti‐GABABR encephalitis, was the most commonly used anti‐cancer treatment among the lung cancer patients, with 78.95% having received it alone or in combination with radiation therapy/surgery, resembling the treatment modalities frequently used in the West (Boronat et al., 2011; Höftberger et al., 2013).

Remarkably, despite the majority of patients having received a combination of corticosteroids and IVIG, the outcome was better in those who were given corticosteroids alone and IVIG alone when compared to those given corticosteroids combined with IVIG. Moreover, the relapse rates of those given corticosteroids alone and IVIG alone were lower than those given corticosteroids combined with IVIG. This could be because the ones who received the combined therapy were far severe in the disease progression and the substantially lesser proportion of the patients receiving monotherapy. Notably, those who received first and second line combined had significantly worse outcome than those who received first line alone. This paradoxical outcome could be explained by the small population in our pool who received this modality in comparison to the first line alone and also because of the severity bias.

The mean duration of follow‐up was 14 months during which relapse was reported in 19.15%; this is in the higher range compared to that reported by Lancaster et al. (2010) and Jeffery et al. (2013). This could be attributed to the shorter duration of follow‐up in these studies (9 months and 1 month, respectively). Moreover, difference between ethnicities has to be ruled out. In our study, 63.6% had favorable outcome (mRS 0–2), which agrees with a previous study (Höftberger et al., 2013), but was strikingly better than the findings reported in other western studies (Boronat et al., 2011; Jeffery et al., 2013; Lancaster et al., 2010). Despite the heterogeneities in the outcome measurement scales and lengths of follow‐up, it is reasonable to suggest the possibility of better outcome in the Asian population.

The mortality rate in our study is consistent with that reported by Jeffery et al. (2013) but lower than most western studies, reflecting lower paraneoplastic rate in Asians and further advocating the possibility of improved outcome in this population (Boronat et al., 2011; Höftberger et al., 2013; Lancaster et al., 2010; Maureille et al., 2019). As expected, the most common cause of death was tumor progression, followed by respiratory failure, pulmonary infections, status epilepticus, and septic shock, which mirrors the findings in other studies (Boronat et al., 2011; Höftberger et al., 2013). Studies have shown that co‐existence of onconeural antibodies against intracellular antigens are refractory to immunotherapy and have worse prognosis (Höftberger et al., 2013; Jeffery et al., 2013). Owing to frequent tumor association and significant morbidity and mortality caused by it, repeated tumor screening in follow‐up, especially in the elderly, is of paramount significance even when the initial presentation is tumor‐free (Graus et al., 2004; Titulaer et al., 2011). Early detection of tumors and aggressive tumor management may even result in better outcome (Maureille et al., 2019).

### 4.3 Anti‐CASPR2 encephalitis

CASPR2 is a membrane protein expressed in the central and peripheral nervous system, particularly in the cortex, limbic system, basal ganglia, brainstem, thalamus, and sensory organs (Gordon et al., 2016). It is a cell adhesion molecule of the neurexin family responsible for synapse formation, regulation, and neuronal network establishment (Horresh et al., 2008; Saint‐Martin et al., 2018). Studies have shown that mutations in the gene encoding CASPR2 lead to focal epilepsy, mental retardation, schizophrenia, and other neuropsychiatric problems (Friedman et al., 2008; Saint‐Martin et al., 2018; Strauss et al., 2006). Hence, comparable clinical syndrome would be justified in patients with anti‐CASPR2 antibodies as well.

The mean age for the patients (47.7 years) and sex ratio (70% males) in our study echoed a recent systematic analysis (Boyko, Au, Casault, de Robles, & Pfeffer, 2020). The tumor prevalence rate has been reported from 0% to 52.2% (Becker et al., 2012; Irani et al., 2010), the majority being thymomas. In our study, no malignancies were seen, but this could be an underestimation because of potential inadequacies in screening, as reported by one of the studies (Sunwoo et al., 2015).

Unlike other forms of autoimmune encephalitides, anti‐CASPR2 variant is associated with a wide range of signs and symptoms which go beyond the central nervous system. In our study, despite the low sample size, symptoms like cognitive defects, epilepsy, peripheral nerve hyperexcitability/neuromyotonia, neuropathic pain, cerebellar symptoms, and dysautonomia presenting either as limbic encephalitis or Morvan's syndrome were noted, reflecting the variability in presentation reported in previous works (Becker et al., 2012; Bien et al., 2017; Boyko et al., 2020; Klein et al., 2013; van Sonderen, Ariño, et al., 2016).

CSF abnormalities have been reported in 35%–40% patients, the commonest changes being pleocytosis (21%) and increased protein concentration (26%), congruent with our report (25% each) (Bien et al., 2017; van Sonderen, Ariño, et al., 2016). About two‐thirds of the patients are reported to have an abnormal EEG with non‐specific findings like slowing and epileptic discharges (Boyko et al., 2020; van Sonderen, Ariño, et al., 2016). All the patients had abnormal EEGs in our study, with similar major findings. Previous reports have stated half the patients to have abnormal MRI findings, MTL, and hippocampus being commonly involved (van Sonderen, Ariño, et al., 2016). MTL involvement was seen in a third of the patients on T2‐weighted MRI in our study. Among the 3 patients whose FDG‐PET scans were available, none showed abnormalities. However, FDG‐PET scans are normally reported to have a much higher abnormality rate, mesiotemporal hypermetabolism being the commonest anomaly (Morbelli et al., 2016). This discrepancy is most likely the result of the small number of patients.

Immunotherapy was used in 90% patients, corresponding to previous studies (van Sonderen, Ariño, et al., 2016). In our study, the relapse rate was the highest among the ones treated with corticosteroids alone. Owing to the small sample size in our study, no concrete conclusion can be possibly drawn regarding the inadequacy of corticosteroids monotherapy. One patient who refused immunotherapy did not show any clinical improvement, whereas the remaining patients who were given immunotherapy had significant improvement including plausible seizure control, consistent with other papers (Irani et al., 2010; Lancaster et al., 2011; Sunwoo et al., 2015). This reflects the favorable response of anti‐CASPR2 encephalitis to immunotherapy (Irani et al., 2010; Lancaster et al., 2011).

### 4.4 Limitations

There was variation in reporting due to potentially different definitions used by different authors, lack of uniform nomenclature and non‐specific terminologies used in several papers, especially regarding psychiatric symptoms. This required author consensus to create plausible categories while entering data. We could not contact some authors and access unpublished/supplementary data. The timing of diagnostic investigations was not reported by many publications and could not be taken into account while interpreting their findings, which is another limitation of the study. Furthermore, only ten cases of anti‐CASPR2 encephalitis could be identified, compromising the reliability of our interpretation.

## 5 CONCLUSION

Our study suggests that autoimmune encephalitis is a treatable condition; early diagnosis and prompt treatment lead to better outcomes. Therefore, a high index of clinical suspicion and better accessibility of autoantibody testing in suspected patients are encouraged. If available, exploration of advanced diagnostic modalities like FDG‐PET could lead to earlier detection of brain changes and subsequently earlier treatment. We found the age at presentation to be younger, the outcome to be better and the mortality to be lesser in the Asian population. To further explore these findings, we emphasize the need for additional epidemiological studies, especially for anti‐CASPR2 encephalitis wherein the available literature is insufficient. A lower prevalence of tumor association was seen, although this could have resulted from inadequate screening. We thus suggest serial malignancy screenings using CT/MRI scans for commonly associated tumors like lung cancers. To address the lack of a standard treatment protocol, multicenter studies with a larger number of patients are warranted in the future comparing various types of immunotherapy, which could result in better treatment outcomes.

## CONFLICT OF INTEREST

None of the authors have any conflicts of interest to disclose.

## AUTHOR CONTRIBUTIONS

RO, UPK, and PG participated in the conceptualization of the study and manuscript preparation. UPK and PG participated in literature search, data extraction, analysis, and writing the manuscript. BPG, RK, RR, SP, and NG participated in manuscript review. RO supervised the research, provided critical intellectual input, and edited the final manuscript. All authors participated in revising the manuscript and approved the final manuscript.

### Peer Review

The peer review history for this article is available at https://publons.com/publon/10.1002/brb3.1793.

## Supporting information

Appendix S1

## Data Availability

The data that support the findings of this study are available from the corresponding author upon reasonable request.
