# Supplementary material for: Anti‐LGI1, anti‐GABABR, and Anti‐CASPR2 encephalitides in Asia: A systematic review
Source: Brain Behav. 2020 Aug 12;10(10):e01793. doi: 10.1002/brb3.1793 (PMC7559615; doi:10.1002/brb3.1793)
Supplement: Supplementary file 1 — Appendix S1 [file BRB3-10-e01793-s001.docx]

**Supporting information: Search Strategy**

**Date of Search**: 24th May, 2020

**Filters Used:**  None

**Search syntax:** (“Term_1” OR “Term_2” OR “Term_3”...) AND (“Name of Country” OR “Capital” OR “City1” OR “City2”...)

**“Term_n”s used were:**

1. Autoimmune Encephalitis
2. Anti LGI1 encephalitis
3. Anti GABAB encephalitis
4. Anti GABABR encephalitis
5. Anti GABAB-R encephalitis
6. Anti CASPR encephalitis
7. Anti CASPR2 encephalitis
8. Anti AMPA encephalitis
9. Anti AMPAR encephalitis
10. Anti AMPA-R encephalitis

**Names of Countries and Cities Used were:**

1. Afghanistan, Kabul
2. Armenia, Yerevan
3. Azerbaijan, Baku
4. Bahrain, Manama
5. Bangladesh, Dhaka
6. Bhutan, Thimphu, Thimpu
7. Brunei, Bandar Seri Begawan
8. Myanmar, Naypyidaw
9. Cambodia, Phnom Penh
10. China, Beijing, Guangzhou, Shanghai, Chongqing, Hangzhou, Wuhan, Chengdu, Tianjin,  Xi’an, Jinan
11. Georgia, Tbilisi
12. Hong Kong, Hong Kong
13. India, Delhi, Mumbai, Bangalore, Hyderabad, Ahmedabad, Chennai, Kolkata, Surat, Pune, Jaipur
14. Indonesia, Jakarta
15. Iran, Tehran
16. Iraq, Baghdad
17. Israel, Jerusalem
18. Japan, Tokyo, Yokohama, Osaka, Nagoya, Sapporo, Fukuoka, Kobe, Kawasaki, Kyoto, Saitama
19. Jordan, Amman
20. Kazakhstan, Nur Sultan
21. North Korea, Pyongyang
22. South Korea, Seoul
23. Kuwait, Kuwait City
24. Kyrgyzstan, Bishkek
25. Laos, Vientiane
26. Lebanon, Beirut
27. Macau
28. Malaysia, Kuala Lumpur
29. Maldives, Male
30. Mongolia, Ulaanbaatar
31. Nepal, Kathmandu
32. Oman, Muscat
33. Pakistan, Islamabad
34. Philippines, Manila
35. Qatar, Qatar
36. Saudi Arabia, Riyadh
37. Singapore, Singapore
38. Sri Lanka, Colombo
39. Syria, Damascus
40. Taiwan, Taipei
41. Tajikistan, Dushanbe
42. Thailand, Bangkok
43. Timor-Leste, Dili
44. Turkmenistan, Ashgabat
45. United Arab Emirates, Abu Dhabi
46. Uzbekistan, Tashkent
47. Vietnam, Hanoi
48. Yemen, Sanaa
